# Gender differences in neck muscle activity during near-maximum forward head flexion while using smartphones with varied postures

**DOI:** 10.1038/s41598-024-63734-0

**Published:** 2024-06-06

**Authors:** Yi-Lang Chen, You-Chun Chan, Hans Alexander

**Affiliations:** 1 Department of Industrial Engineering and Management, Ming Chi University of Technology, 84 Gung-Juan Road, Taishan, New Taipei 243303 Taiwan; 2 Formosa Plastics Group, Taipei, 114030 Taiwan; 3 Apical Group, Singapore, 048624 Singapore

**Keywords:** Smartphone user, Gender difference, Neck flexion, Muscle activity, Head angle, Health care, Risk factors

## Abstract

Women frequently express heightened neck discomfort even though they exhibit smaller neck flexion (NF) during smartphone use. Differences in natural posture while using smartphones may result in varying muscle activation patterns between genders. However, no study focused on this issue. This study investigated the influence of gender on neck muscle activity and NF when using smartphones, ranging from slight (20°) to nearly maximal forward head flexion, across different postures. We analyzed smartphone usage patterns in 16 men and 16 women and examined these behaviors across different scenarios: standing, supported sitting, and unsupported sitting, at 20°, 30°, 40°, and the maximum head angles. During data collection, muscle activity was measured, expressed as a percentage of the maximum voluntary contraction (%MVC), in the cervical erector spinae (CES) and upper trapezius (UTZ), along with NF. Results show significant influences of gender, head angle, and posture on all measures, with notable interactions among these variables. Women displayed higher muscle activities in CES and UTZ, yet exhibited lesser NF, while using smartphones in both standing (12.3%MVC, 10.7% MVC, and 69.0°, respectively) and unsupported sitting (10.8%MVC, 12.3%MVC, and 71.8°, respectively) compared to men (standing: 9.5%MVC, 8.8%MVC, and 76.1°; unsupported sitting: 9.7%MVC, 10.8%MVC, and 76.1°). This study provides a potential rationale for gender-related disparities in injury outcomes, emphasizing that women experience higher neck and shoulder discomfort level, despite their smaller NF during smartphone use, as found in previous research. Additionally, the cervical flexion-relaxation phenomenon may occur when the head angle exceeded 40°. The near-maximum head angle during smartphone use might induce the cervical flexion-relaxation phenomenon, potentially aggravating neck issues. We recommend limiting smartphone usage postures that exceed the near-maximum head angle, as they are commonly adopted by individuals in the daily smartphone activities.

## Introduction

In today's modern world, smartphones have seamlessly integrated into our daily routines, playing essential roles in both our personal and professional lives. Surveys conducted across various countries consistently show an increasing trend in the time individuals spend using smartphones each day, with an average of at least 2.4 h, and many users exceeding 5 h per day^1–3^. Whether people are standing, sitting, or anywhere in between, they are almost always connected to the world through their smartphones. While smartphones have brought numerous conveniences to our lives, they have also introduced persistent challenges, with one of the most significant being the physical strain they can impose on our bodies.

The use of smartphones can give rise to various physical issues, with one of the primary concerns being the strain in the neck and shoulder region^4–6^. This connection has been consistently confirmed by surveys conducted in different countries and regions^7–10^. Repetitive neck flexion (NF) while using smartphones has been identified as a risk factor for neck and shoulder pain^11–13^ due to the static load imposed on the relevant muscles and bones^14^. Addressing neck and shoulder pain requires an understanding not only of neck posture but also the associated muscle and nerve changes that occur during NF^15^. NF is closely linked to the activation of specific neck-related muscles during smartphone use, notably the cervical erector spinae (CES) and upper trapezius (UTZ) muscles^16–18^. While there is a consensus in most studies that NF leads to a temporary activation of CES and UTZ, some research has reported an intriguing finding where NF increased CES activity but decreased UTZ activity^19,20^. This contradictory effect may be attributed to the unique demands placed on shoulder muscles when holding a phone, potentially masking the overall impact of tasks on the muscles^21^.

The flexion relaxation phenomenon (FRP), initially described by Floyd and Silver^22^ in 1955, refers to the momentary pause in CES muscle activity after reaching a specific degree of NF^23^. Although typically used to evaluate lumbar muscle activity, this concept is also applicable to the cervical spine^24,25^, making it a valuable tool for assessing neck pain and measuring treatment outcomes. In essence, cervical FRP is recognized as the brief interruption of CES muscle activity during deeper forward NF^23,25,26^. Furthermore, FRP has also been observed in the UTZ muscles^27^. FRP occurs due to passive tissues that can support the joint, leading to a temporary loss of muscle activity within the range of motion. In recent years, the widespread use of smartphones has led to prolonged forward bending of the neck. Investigating the link between this posture and FRP can provide insights into potential neuromuscular mechanisms contributing to neck pain. Although limited research has focused on this topic, Shin and Kim^28^ found that measurements related to FRP could have a significant impact on the neuromuscular issues experienced by individuals with chronic neck pain. However, they also acknowledged the challenge of differentiating between neck pain caused by short-term smartphone use, emphasizing the need for further investigation.

Moreover, individuals often unknowingly adopt extreme forward head flexion postures while using smartphones, ranging approximately from 33° to 45°^11,29^. This larger or near-maximal head flexion places strain on the neck extensor muscles through FRP. However, prior research primarily focused on maximum forward head flexion^28,30^, leaving gaps in understanding how FRP evolves from a normal head position to maximum forward flexion. It is crucial to elucidate this relationship between varying head forward positions and the neck muscle activities in smartphone users, especially in identifying when FRP occurs.

Previous studies have consistently shown that women using mobile electronic devices like smartphones are more likely to experience discomfort in the neck and shoulder region compared to men^8,31,32^. Surprisingly, despite women reporting more discomfort, earlier research consistently indicates that women tend to bend their neck forward less than men^16,33–35^. As previously noted, NF is the primary indicator associated with neck discomfort^4^. This discomfort is also linked to the concept of phubbing and has been found to increase the risk of neck disorders following 12 months of smartphone usage, with an odds ratio of 2.44. While Guan and his colleagues^33^ were the first to investigate gender differences in head and neck posture when using smartphones, they did not collect data on the activities of neck-related muscles in their study. However, a paradoxical phenomenon persists: despite women exhibiting lower levels of NF, they experience a higher prevalence of neck discomfort. An alternative explanation for this inconsistency could be the inherent differences in postures adopted by men and women^36^. These differences might lead to variations in neck and shoulder muscle activity between genders during smartphone use. Although recent studies have explored the relationship between smartphone use and neck muscle activity^19–21^; however, these investigations did not specifically address gender differences.

Expanding on the gaps identified in previous research, particularly regarding the impact of forward head angle (HA) on the FRP of neck muscles, as well as the contradictory results concerning gender disparities in the prevalence of neck discomfort and NF, this study aims to address these gaps. In this study, we assessed the activities of the CES and UTZ muscles across various conditions while participants used smartphones. These conditions encompassed standing, sitting with or without back support, and at four distinct HAs (20°, 30°, 40°, and the maximum HA). The NFs were also measured. The study objective was to understand the reasons behind the differing rates of neck and shoulder discomfort experienced by men and women while using smartphones. We also explored how changes in the FRP related to different smartphone usage scenarios and head flexion angles. Our hypothesis was that differences in muscle activity were linked to gender-specific postural characteristics, and various FRP patterns could help explain the strain on the neck and shoulders in different smartphone usage situations.

## Methods

### Participants

In this cross-sectional study, we enrolled a total of 32 young smartphone users, evenly distributed with 16 men and 16 women. All participants reported no history of musculoskeletal disorders, and they met certain criteria. To be part of the study, they needed to have been using a smartphone for at least one year and spent at least three hours per day on their smartphones. The male participants had an average (standard deviation) age of 22.1 (0.8) years, a height of 170.5 (5.2) cm, and a body mass of 65.7 (8.7) kg. In contrast, the female participants had an average (standard deviation) age of 20.9 (1.0) years, a height of 160.8 (5.1) cm, and a body mass of 55.8 (7.6) kg, as shown in Table 1. The study obtained approval from the Ethics Committee of National Taiwan University, Taiwan (Code: NTU-REC 202012EM025), and all methods were carried out in accordance with the relevant guidelines and regulations of the 2013 World Medical Association Declaration of Helsinki. Informed consent was obtained from all participants.

**Table 1:** Anthropometric data of the study participants.

Items	Males (n = 16)		Females (n = 16)	
	Mean (SD)	Range	Mean (SD)	Range
Age (years)	22.1 (0.8)	21–25	20.9 (1.0)	19–22
Height (cm)	170.5 (5.2)	161–177	160.8 (5.1)	154–171
Body mass (kg)	65.7 (8.7)	54–80	55.8 (7.6)	48–72
Body mass index (kg/m^2^)	22.5 (3.1)	21–27	21.3 (39)	18–29
Maximum head angle (°)	47.5 (5.2)	43–55	48.3 (6.1)	43–54

SD, standard deviation.

### Head angle and neck flexion

**Figure 1:**
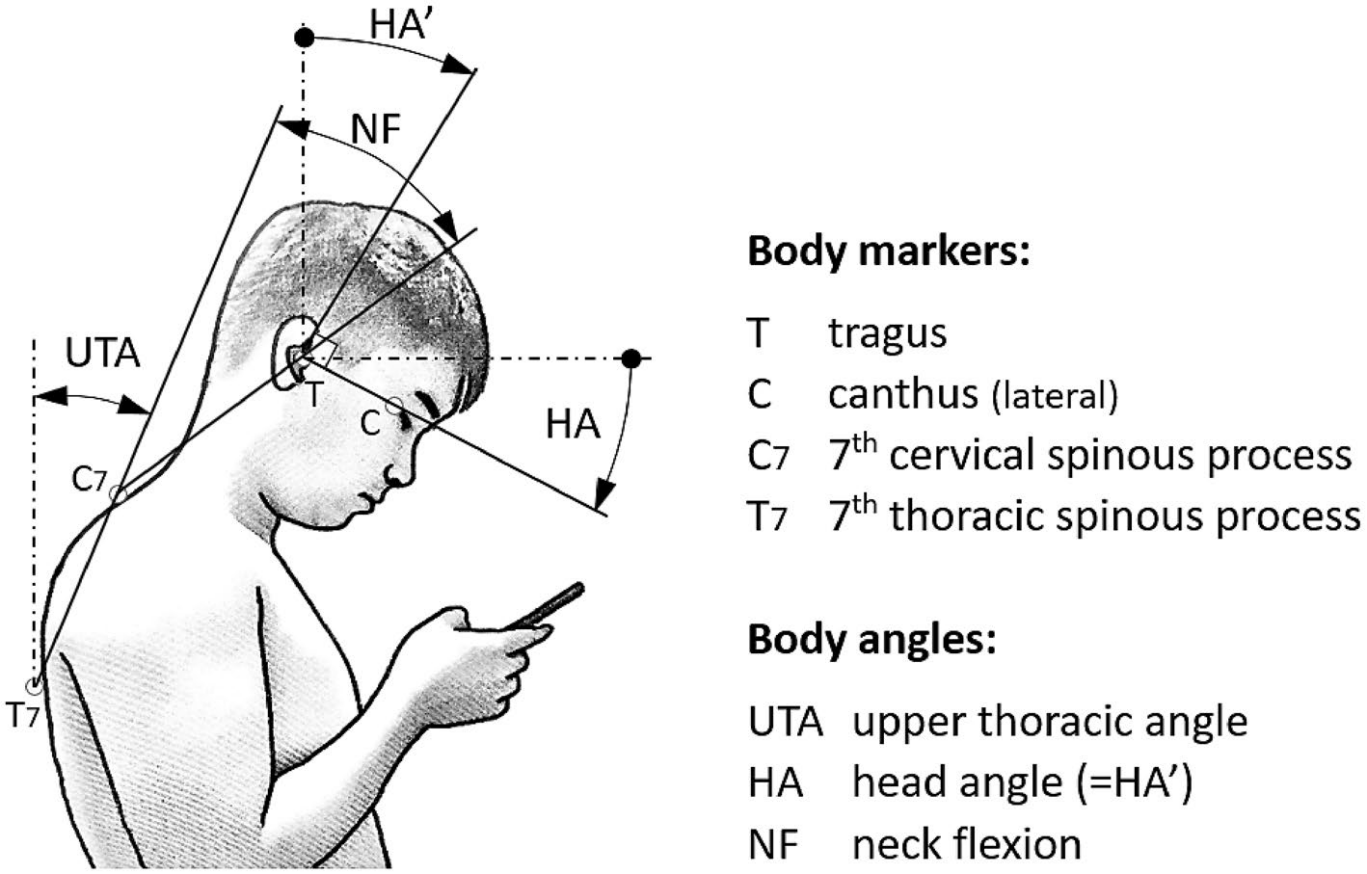
Schematic of the markers and definitions of angles on the human body.

In this study, we manipulated the HA positions in four different angles in the sagittal plane, and utilized the MacReflex motion analysis system (Qualisys, Gothenburg, Sweden) to record the NF of participants in various smartphone-use postures. Biomechanically, NF typically encompasses the forward flexion of both the head and cervical spine as a single unit, while HA specifically refers to the bending of the head with the upper cervical spine serving as the axis of rotation. The mechanisms of forward head and neck flexion indeed differ fundamentally^37,38^. To ensure accurate calculations of NF, previous studies have utilized the upper thoracic angle (UTA) as a reference^21,35^. The UTA is the angle formed between the line connecting C7 to T7 and a vertical line while the participant was either standing or sitting in their natural posture. Since the upper thoracic spine is considered rigid, UTA is a reliable baseline for NF measurements. Notably, the link between UTA and NF is more noticeable in a seated position than in a standing one^21,39^. Figure 1 illustrates the relationship between HA, NF, and UTA, where in the figure, HA’ = HA.

Prior to data collection, we attached four adhesive reflective markers to specific body parts: the lateral canthus (C), tragus (T), seventh cervical spinous process (C7), and thoracic spinous process (T7). Data collection involved participants adopting various postures, including standing and sitting, both with and without back support, while bending their heads at angles of 20°, 30°, 40°, and their maximum HA. The chosen HAs were in line with common user habits, as smartphone users typically maintain an HA of at least 30° from the vertical when using their devices^11,40^. Therefore, angles of 0° and 10°, as set in previous studies, were not examined. These angles were also confirmed based on pilot testing, as all participants could comfortably achieve HAs exceeding 40°, while approximately half of them couldn't reach 50°. When performing the maximum HA, participants were required to bend their heads forward as far as possible without experiencing any discomfort. As indicated in Table 1, the maximum HA achieved by male and female participants was 47.5° and 48.3°, respectively, revealing no significant difference between the two genders.

### Muscle activation

In Fig. 1, we utilized a TeleMyo 2400 electromyograph (EMG) from Noraxon (Scottsdale, AZ, USA) to examine the CES and UTZ muscle groups on the dominant side of each participant. All procedures, including EMG testing (skin preparation, electrode placement, and electrode fixation), data acquisition, and data processing, were conducted in accordance with the guidelines set forth by the Surface EMG for the Non-Invasive Assessment of Muscles (SENIAM) project^41–43^. We placed a pair of Ag/AgCl surface electrodes (with a lead-off area of 10 × 10 mm^2^ and an inter-electrode distance of approximately 20 mm) parallel to the target muscles. However, it is essential to note that we followed SENIAM recommendations by preparing the skin through shaving and alcohol cleaning before electrode attachment. The placement of electrodes for measuring CES and UTZ EMG followed established patterns outlined in previous studies. Specifically, CES electrodes were positioned laterally to the spinous processes, approximately 2 cm from the C4 vertebra, targeting the muscular prominence of the semispinalis and splenius capitis^28,44^. UTZ electrodes were positioned just lateral to the muscle belly along the ridges of the shoulders, midway between the spinous process of C7 and the lateral edges of the acromion^45^.

Prior to conducting EMG measurements, participants were instructed to perform standardized maximum voluntary contraction (MVC) exercises for muscle-specific normalization of the EMG data collected in each trial, in line with the protocol outlined by Vera-Garcia et al.^46^. For the MVC assessments, participants were seated. The MVC of the CES was assessed through resisted neck extension, while the MVC of the UTZ was determined by resisted shoulder elevation, as described in previous studies^16,19^. Participants were instructed to exert their maximal effort (> 2 s) to resist neck extension and shoulder elevation against single and two fixed straps, respectively, with each MVC test lasting for a gradual exertion duration of 5 s. Each participant completed three trials of resisted exertion for each muscle group. To prevent muscle fatigue, each MVC test for the same muscle group entailed maintaining maximum exertion for more than 2 s^44^, followed by a rest period of at least 10 s before the next trial. Additionally, there was a 5 min break before testing the other muscle group, adhering to established MVC testing protocols by Greig et al.^44^ and Namwongsa et al.^19^. The maximum EMG amplitude of each muscle across MVC techniques was calculated using a 0.5 s moving average window^46^, and the highest recorded value from the three trials was utilized as the MVC value for subsequent analysis^47^. The electrical signals collected from both the MVC tests and the experimental trials were subjected to band-pass filtering (20–600 Hz) and sampled at a rate of 1200 Hz^41,43^. Integrated EMG (IEMG) data was derived by rectifying and processing the sampled signals. A normalization procedure was applied to compare the IEMG data from the experimental trial with the MVC IEMG data for a matching 2 s interval. All muscle activation values are expressed as percentages of the MVC IEMG data.

### Experimental design and procedure

In this investigation, we conducted 36 trials concerning smartphone usage, which encompassed four different HAs, three distinct postures, and three repetitions in order to gather data on NF, CES, and UTZ EMG data from our study participants. To ensure data consistency, we calculated the average results of the two closest measurements from the three repetitions for each variable. The four HA positions tested were 20°, 30°, 40°, and the maximum, while the three postures included standing, sitting with back support, and sitting without back support. To maintain consistent HAs across these postures, we established the HA reference point as 0° by using the UTA that each participant naturally assumed when standing, aligning with prior studies by Yoon et al.^21^ and Chen et al.^35^.

Throughout each trial, participants were allowed to use their personal smartphones and were tasked with responding to predetermined questions sent individually via LINE (Z Holdings, Tokyo, Japan) by an experimenter. These questions encompassed basic participant information, including sex, age, height, and body mass, as well as daily topics such as the weather and recent news. The initial HA setting for each participant was randomly selected, and we followed a counterbalanced design, meaning that if the initial HA was set at 40°, subsequent tests occurred in the following sequence: maximum HA, 20°, and 30°. Each trial consisted of a 1.5 min testing period, with data collection for NF, CES EMG, and UTZ EMG taking place during the final 10 s. To ensure data accuracy and prevent participant fatigue, a minimum of 2 min of rest was required between each trial. During rest intervals, participants were instructed to relax in a chair with neck support. Furthermore, Thorburn et al.^2^ discovered that musculoskeletal discomfort resulting from smartphone use typically occurred within 15 to 30 min after use.

The temporal arrangement closely followed the experimental protocols outlined by Namwongsa et al.^19^. It was assumed that the data for the dependent variable remained relatively stable and consistent during the final 10-s intervals, allowing an adequate timeframe to assess the shoulder and neck load generated by texting with smartphones using both hands, with a fixed specific HA. The decision to set the duration of each testing trial at 1.5 min was primarily guided by the aim to enable direct comparisons with similar studies. Specifically, Namwongsa et al.^19^ employed a trial duration of 1.5 min in their study, while another related study conducted by Yoon et al.^21^ utilized a shorter duration of 1 min for their trials. Typically, each participant underwent three testing sessions with repetitions, and each session lasted less than 1.5 h during a half-day period.

For the tests, we positioned a MacReflex motion analysis system (Qualisys) approximately 5 m away from the right side of each participant, aligned perpendicular to the sagittal plane. This arrangement was utilized to record two-dimensional marker positions. Participants were directed to naturally tilt their heads in the sagittal plane during the test. To ascertain the correct HA, except for the individual maximum HA, each participant was requested to align their line of sight (the line connecting between the markers of lateral canthus and tragus) with a reference line displayed on the motion analysis system's feedback monitor. Once the HA matched the desired position, participants were instructed to maintain that posture (e.g., NF) as they would normally.

### Statistical analysis

We performed data analysis using IBM SPSS Statistics version 23.0 (IBM, Armonk, NY, USA) and set the statistical significance level at 0.05 for all statistical tests. To investigate the effects of gender, posture, and HA on muscle EMG and NF data, we employed a three-way analysis of variance (ANOVA). In cases where a significant interaction was observed between two factors, we further conducted two-way ANOVA. Multiple comparisons were carried out using Duncan's multiple range test. Effect sizes were quantified using η^2^ values for each effect, as outlined by Cohen^48^. Prior to the analysis, we assessed the normal distribution of numerical variables using the Kolmogorov–Smirnov test and confirmed the homogeneity of variances through Levene's test.

### Ethics approval

This research was approved by the Ethics Committee of National Taiwan University, Taiwan (protocol code NTU-REC 202012-EM-025) and was conducted according to the guidelines of the Declaration of Helsinki. Other ethical criteria included written consent to participate in the study and withdraw from the study whenever participants were willing.

## Results

### Three-way ANOVA results

Table 2 displays the outcomes of the three-way ANOVA, with gender, posture, and HA as the independent variables. Notably, significant main effects of these variables on the measured responses were observed. Specifically, during each interaction, gender and posture were found to significantly affect two muscle EMGs, while posture and HA significantly affected UTZ EMG and NF. Given that all three variables were observed to interact with each other, a cross-analysis was deemed necessary.

**Table 2:** Three-way ANOVA result for three measured variables.

Variables	Responses	SS	DF	MS	F	Significance	η^2^
Gender (G)	Cervical erector spinae	150	1	150	21.47	< 0.001	0.060
	Upper trapezius	107	1	107	12.74	< 0.001	0.037
	Neck flexion	2408	1	2408	54.87	< 0.001	0.140
Posture (P)	Cervical erector spinae	50	2	25	3.61	< 0.05	0.021
	Upper trapezius	284	2	142	16.91	< 0.001	0.091
	Neck flexion	330	2	165	3.77	< 0.05	0.024
Head angle (HA)	Cervical erector spinae	621	3	207	29.68	< 0.001	0.209
	Upper trapezius	579	3	193	22.94	< 0.001	0.170
	Neck flexion	41306	3	13769	313.76	< 0.001	0.737
G × P	Cervical erector spinae	123	2	61	8.81	< 0.001	0.050
	Upper trapezius	70	2	35	4.14	< 0.05	0.024
	Neck flexion	170	2	85	1.93	0.147	0.011
G × HA	Cervical erector spinae	27	3	9	1.28	0.282	0.011
	Upper trapezius	27	3	9	1.07	0.360	0.010
	Neck flexion	356	3	119	2.70	< 0.05	0.024
P × HA	Cervical erector spinae	29	6	5	0.69	0.658	0.012
	Upper trapezius	221	6	37	4.39	< 0.001	0.073
	Neck flexion	800	6	133	3.03	< 0.01	0.051
G × P × HA	Cervical erector spinae	26	6	4	0.63	0.704	0.011
	Upper trapezius	39	6	6	0.76	0.598	0.013
	Neck flexion	28	6	5	0.11	0.996	0.002

SS, Sum of square; DF, Degree of freedom; MS, Mean square; F, F-value; η^2^, Effect size.

### Cross-analysis for interaction

**Figure 2:**
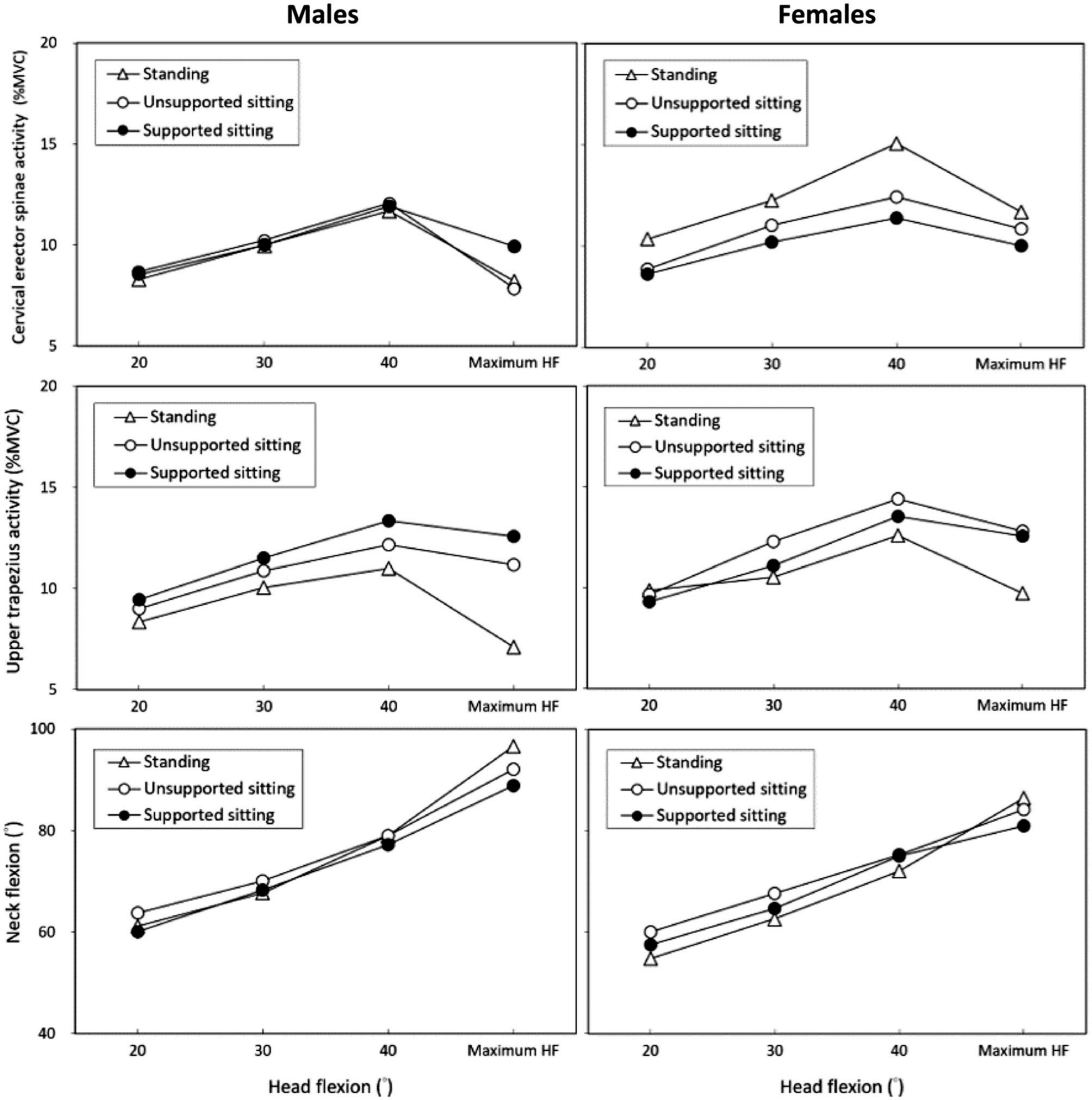
Comparisons of measured variables among male and female participants under different head angles and three postures.

**Figure 3:**
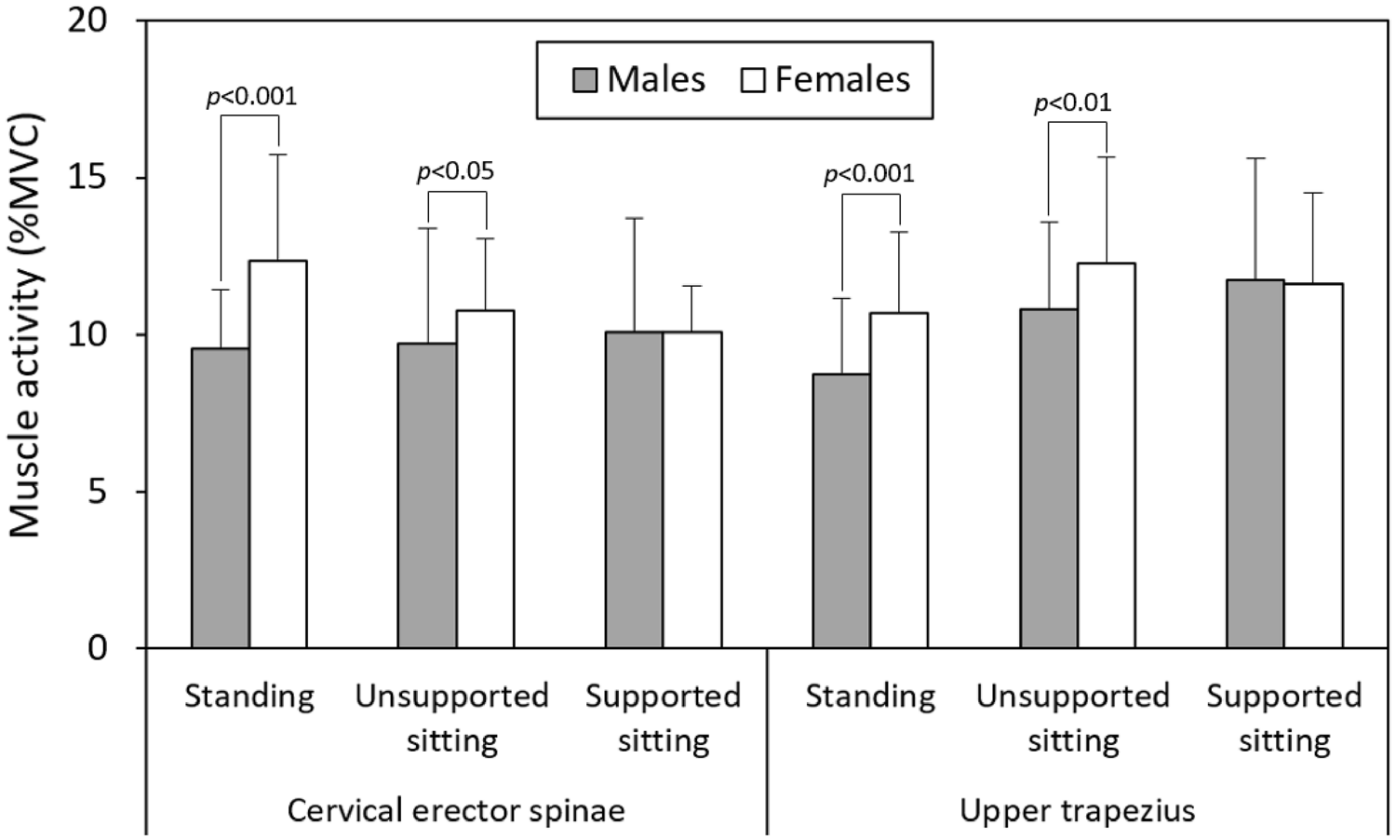
Comparisons of cervical erector spinae and upper trapezius activities between genders across three postures.

Table 3 presents the results of the two-way ANOVA, analyzed with respect to the posture and HA variables for each gender, while Tables 4 and 5 provide details of the multiple comparisons. In Table 3, it is evident that HA consistently affected all three measures, but the posture variable exhibited different trends in CES EMG and NF for both genders. Among the three postures, women demonstrated variations in CES EMG, whereas men showed differences in NF. Specifically, when standing for smartphone use, women displayed a relatively high CES EMG (12.34%MVC), while men exhibited a lower EMG in UTZ (8.75%MVC) compared to other postures (Table 4). Among the different HAs, as shown in Table 5, all the measures progressively increased when HA changing from 20° to 40°. However, the EMG values of the two muscle groups dramatically decreased at the maximum HA for both genders. Figure 2 provides additional insight into the cross-analyzed results. It demonstrates notable distinctions in the EMG values of two muscles across three different postures, distinguishing between genders. Furthermore, the figure provides further evidence of the FRP in the muscles associated with the cervical region. It is important to highlight that, except in the case of supported sitting, women consistently exhibited significantly higher levels of muscle activity in comparison to men (Fig. 3).

**Table 3:** Two-way ANOVA results analyzed by posture and head angle for each gender.

Gender	Variables		Responses	SS	DF	MS	F	Significance	η^2^
Men	Posture		Cervical erector spinae	10	2	5	0.54	0.582	0.006
			Upper trapezius	277	2	139	18.32	< 0.001	0.179
			Neck flexion	247	2	124	3.32	< 0.05	0.038
	Head angle		Cervical erector spinae	338	3	113	12.26	< 0.001	0.180
			Upper trapezius	264	3	88	11.63	< 0.001	0.172
			Neck flexion	24010	3	8003	214.98	< 0.001	0.793
Women	Posture		Cervical erector spinae	163	2	82	17.17	< 0.001	0.170
			Upper trapezius	77	2	39	4.17	< 0.05	0.047
			Neck flexion	253	2	126	2.50	0.085	0.029
	Head angle		Cervical erector spinae	309	3	103	21.71	< 0.001	0.279
			Upper trapezius	342	3	114	12.32	< 0.001	0.180
			Neck flexion	17652	3	5884	116.43	< 0.001	0.675

SS, Sum of square; DF, Degree of freedom; MS, Mean square; F, F-value; η^2^, Effect size.

**Table 4:** Multiple comparisons of varying postues for each gender.

	Postures	Cervical erector spinae (%MVC)	Upper trapezius (%MVC)	Neck flexion (°)
Men	Standing	9.53 (5.79–13.27)^a^	8.75 (3.93–13.57)^a^	76.1 (47.3–104.9)^a^
	Unsupported sitting	9.69 (2.40–16.98)^a^	10.80 (5.29–16.31)^b^	76.1 (52.0–100.2)^a^
	Supported sitting	10.09 (2.94–17.24)^a^	11.72 (4.04–19.40)^b^	73.6 (49.7–97.5)^b^
Women	Standing	12.34 (5.66–19.02)^a^	10.67 (5.04–15.90)^a^	69.0 (42.0–96.0)^a^
	Unsupported sitting	10.77 (8.25–13.29)^b^	12.27 (5.59–18.95)^b^	71.8 (49.3–94.3)^a^
	Supported sitting	10.06 (7.12–13.00)^b^	11.60 (5.76–17.44)^ab^	69.6 (47.8–91.4)^a^

Data (mean, with 95% confidence interval in parentheses) with the same letter do not differ in the Duncan test.

**Table 5:** Comparisons in all responses of varying head angles for each gender.

	Head angle (°)	Cervical erector spinae (%MVC)	Upper trapezius (%MVC)	Neck flexion (°)
Men	20	8.49 (5.06–11.92)^a^	8.93 (3.64–14.22)^a^	61.6 (51.2–72.0)^a^
	30	10.05 (4.05–16.05)^b^	10.81 (5.36–16.26)^b^	68.7 (57.5–79.9)^b^
	40	11.89 (3.09–20.69)^c^	12.18 (5.22–19.14)^c^	78.4 (64.3–92.5)^c^
	Maximum	8.63 (5.09–12.17)^a^	9.78 (3.86–15.70)^ab^	92.4 (79.5–105.3)^d^
Women	20	9.27 (5.12–13.42)^a^	9.61 (4.61–14.61)^a^	57.4 (41.7–73.1)^a^
	30	11.16 (6.28–16.04)^b^	11.30 (6.03–16.57)^b^	65.0 (49.3–80.7)^b^
	40	12.96 (6.96–18.96)^c^	13.50 (4.60–22.40)^c^	74.2 (60.5–87.9)^c^
	Maximum	10.84 (7.84–13.86)^b^	11.66 (7.90–15.42)^b^	83.9 (73.1–94.7)^d^

Data (mean, with 95% confidence interval in parentheses) with the same letter do not differ in the Duncan test.

## Discussion

The use of smartphones has become an integral and ubiquitous aspect of modern life for people. Whether they are standing or sitting, in private spaces or public areas, it has become commonplace to use smartphones at any time and in any place. Different postures while using smartphones negatively affect the users' NF and the associated muscle activity^17,18,21,49^. Previous research on gender differences in neck and shoulder strain during smartphone use has yielded inconsistent results. For instance, women generally exhibited a higher prevalence of discomfort in their neck and shoulders compared to men^8,31,32^, but studies have also indicated that women have significantly smaller NF when using smartphones^16,33–35^. This study hypothesized that this contradiction may be due to inherent posture differences between genders, and the findings of this study also support this hypothesis.

In the results, although women have smaller NF (see Tables 2 and 4), their neck-related muscle activities are relatively higher than men, especially when standing or sitting without support (see Fig. 3), which may partially explain the higher prevalence of neck and shoulder discomfort among women. As depicted in Fig. 3, except for supported sitting, female participants consistently exhibited significantly higher activities in neck and shoulder muscles. The differences in CES and UTZ EMG values between genders when standing were 2.81%MVC (*p* < 0.001) and 1.92%MVC (*p* < 0.001), respectively, while the corresponding differences during unsupported sitting were 1.08%MVC (*p* < 0.05) and 1.47%MVC (*p* < 0.01). This study offers a potential explanation for gender-related disparities in injury outcomes, highlighting women's heightened susceptibility to neck and shoulder discomfort when using smartphones, as observed in previous investigations^8,31,32^. The findings also indirectly emphasized that while NF is associated with neck strain during smartphone use, it might not be the sole determining factor.

While both men and women tend to maintain similar habitual postures while standing, it is noteworthy that men often adopt a considerably more forward-flexed neck position when using their smartphones in the upright position^33^. Additionally, when sitting, men tend to maintain a more flexed posture, in contrast to the posture commonly observed in women^16^. However, as previously mentioned, women generally report a higher prevalence of discomfort in their neck and shoulders compared to men. While some researchers have suggested that this may be due to women having higher pain sensitivity^50^ or different computer usage habits^51^, these general explanations may not fully account for the higher discomfort in the neck and shoulder reported by women while using smartphones compared to men. In the analysis, as shown in Tables 2 and 4, it was observed that women consistently maintained an upright neck posture for all three positions (Fig. 2), whereas men tended to adopt a more relaxed posture. These findings are consistent with the research conducted by Korakakis et al.^36^, which also discovered that women tend to maintain more upright postures than men when instructed to sit in their optimal positions. In our current study, a similar trend was observed, especially in the standing position. It is worth noting that supported sitting, as suggested by previous studies, has the potential to reduce the NF angle and alleviate strain on the extensor muscles of the cervical spine, particularly the CES muscles^19,20^. However, in our investigation, this effect was only significant in males for NF when they assumed the supported seated position (Table 4). While differences in NF between genders may be influenced by inherent anthropometric variations, such as body height and arm length, it is important to emphasize that Brink et al.^52^ did not find any significant correlation between male or female height and body angle measurements. The observed gender differences in body angles, such as NF, may be reasonably attributed to distinct postural characteristics.

In this study, it was observed that as NF approached its maximum during smartphone use, the cervical FRP might take over the role of cervical extensor muscles, such as the CES, responsible for facilitating forward bending with passive tissues in proximity to the cervical spine^23^. Passive tissues, notably the posterior spinal ligament, step in to counterbalance the moment created by the forward flexion of the head, thus allowing for neck forward flexion. However, this tendency can potentially result in higher loading on ligament tissues, leading to increased cervical spinal loading due to the shorter moment arm of ligaments compared to muscular tissue, as demonstrated by Pialasse et al.^24^ and Gauns and Gurudut^53^. Moreover, ligamentous tissues possess viscoelastic properties, implying that the extensor moment provided by these tissues and passive tissue deformation may result from prolonged or repetitive exposure^54^. This study has revealed that FRP is most frequently observed when the HA exceeds 40°, based on the specific HA levels we examined. Therefore, it is advisable to limit the adoption of mobile phone postures that surpass this angle or approach its maximum limit. Unfortunately, Lee et al.^11^ found that smartphone users frequently tilt their heads forward at angles ranging from approximately 33° to 45°. Additionally, Ning et al.^29^ discovered that some smartphone users tend to incline their heads at an angle of 44.7° while using their smartphones.

While different genders and usage postures can contribute to FRP, our study has revealed that various factors can influence the measured responses, as demonstrated in Fig. 2. When women stand and use smartphones, their CES EMG levels were significantly higher than when they were sitting, and there was also a relative increase in the degree of FRP. Furthermore, the FRP in UTZ EMG when standing was more pronounced than in the two sitting postures. It is important to note that, while several studies have failed to observe FRP in the UTZ^26,55,56^, Shin et al.^27^ did find FRP in the UTZ when examining overhead work. Smartphone usage while seated often results in higher NF compared to using smartphones in a standing position, as suggested by several prior studies^11,28,49,57,58^, our study results surprisingly showed no apparent differences among the various postures (Table 4 and Fig. 2). This discrepancy may be attributed to variations in experimental settings among studies and the utilization of UTA for normalizing the NF data in this study.

This study comes with several noteworthy limitations. Firstly, while the results provided valuable insights and the effect sizes, as indicated by η^2^, were satisfactory, the sample size, comprising only 32 young men and women, was relatively small. Secondly, it's essential to acknowledge that the study's generalizability is limited by the narrow age range of the male and female participants, and the fact that the participants did not experience neck pain also restricts the extrapolation of findings to individuals with neck pain or other specific conditions. Therefore, when interpreting the results and applying them to broader populations, these limitations should be taken into account. Furthermore, this study solely focused on static standing and sitting postures during smartphone use. Dynamic activities such as walking can have distinct effects on cervical spine movement, and this aspect warrants further investigation. In our test, participants were instructed to text using Line messages for 1.5 min during each testing trial, with data from the last 10 s utilized for analysis. We assumed that no fatigue effect occurred during this duration, which could reasonably reflect the relevant shoulder and neck load of the testing combination. Muscle activities were assessed only on the participants' dominant side in the study, and the impact of asymmetry effect was not included. Additionally, no HAs between 40° and the maximum were tested. Based on the result, it is reasonable to assume that FRP may occur whenever the HA exceeds 40°. Finally, including a self-selected preferred HA as a test angle may be comparable and meaningful for future investigations in line with the results of this study.

## Conclusions

The problem of neck discomfort and smartphone-related injuries is a significant concern in our digital age. This study investigated the gender-specific aspects of these issues. Our findings suggest that women often exert greater strain on their neck and shoulder muscles, particularly when they stand or sit without back support, which likely contributes to their heightened discomfort when using smartphones in these areas. Additionally, our results indicate that excessive forward head flexion (e.g., a near-maximum position) while using a smartphone can trigger the cervical flexion-relaxation phenomenon, which may worsen neck problems. By examining these factors, this research aims to help better understand the causes of smartphone-related neck issues and find ways to reduce them, especially considering the differences between men and women.

## Data Availability

The datasets generated during and/or analysed during the current study are available from the corresponding author on reasonable request.
